# Lower urinary tract symptoms are elevated with depression in Japanese women

**DOI:** 10.1111/luts.12478

**Published:** 2023-03-30

**Authors:** Sahoko Ninomiya, Takashi Kawahara, Sohgo Tsutsumi, Hiroki Ito, Kazuhide Makiyama, Hiroji Uemura

**Affiliations:** ^1^ Departments of Urology and Renal Transplantation Yokohama City University Medical Center Yokohama Japan; ^2^ Department of Urology Yokohama City University Graduate School of Medicine Yokohama Japan

**Keywords:** depression, Japanese women, lower urinary tract syndrome, questionnaire

## Abstract

**Objectives:**

Depression might worsen lower urinary tract symptoms (LUTS), but the correlation is still disputed. This study examined the influence of depression on LUTS in Japanese women.

**Methods:**

This study used a web‐based questionnaire to evaluate the mental status of depression and LUTS. The mental status of depression was evaluated using the Quick Inventory of Depressive Symptomatology‐Japanese version (QIDS‐J), and LUTS was assessed based on the Overactive Bladder Symptom Score (OABSS) and responses to the International Consultation on Incontinence Questionnaire‐Short Form.

**Results:**

A total of 4151 of 5400 (76.9%) women responded to the questionnaire. The mean age was 48.3 ± 13.8 years. The OABSS gradually increased with the QIDS‐J score. The incidence of overactive bladder (OAB) and urgency urinary incontinence (UUI) also increased along with the QIDS‐J score. In the younger age group (20–39 years old), the risks of OAB and UUI were higher than in the elderly group (7.42 for OAB and 7.44 for UUI).

**Conclusions:**

This study revealed that worsening of LUTS was correlated with depression.

## 1 INTRODUCTION

Lower urinary tract symptoms (LUTS) are divided into voiding symptoms, storage symptoms, and post‐micturition symptoms.^1^ Irwin et al. reported that LUTS, as defined by the International Continence Committee in 2002, were present in 62.5% of men and 66.6% of women aged ≥40 years.^2^ A total of 59.2% of women have been reported to have storage symptoms, including stress urinary incontinence (SUI) and overactive bladder (OAB) symptoms. Zhang et al. also reported that 55.5% of men had LUTS, including 53.9% with storage symptoms, namely 23.4% with nocturia, 23.3% with urgency, and 18.9% with SUI.^3^ In Japanese cohorts, our previous study revealed that 21.4% of women had urgency symptoms, and 16.7% had SUI.^4^

While LUTS are not life‐threatening, they reduce quality of life. In contrast, suicide is strongly correlated with depression, making depression a life‐threatening disease.^5^, ^6^ A total of 264 million people are estimated to have depression globally, accounting for approximately 4.4% of the entire global population.^7^, ^8^ A recent study suggested that the prevalence of depression is double the actual reported values, and the prevalence of depression in women is twice that in men.^8^

Requel et al. revealed that elderly women with depression had a particularly high risk of developing LUTS.^8^ However, Melville et al. noted no correlation between LUTS and depression.^9^ The correlation between depression and LUTS therefore remains controversial. No large study has yet reported on the relationship between depression and LUTS in Japanese women.

The present study therefore investigated the relationship between depression symptoms and LUTS in Japanese women using a web‐based questionnaire.

## 2 MATERIALS AND METHODS

We conducted a web‐based questionnaire analysis using 4.5 million Asian Japanese panels with an internet research company (Freeasy; iBRIDGE, Tokyo, Japan). We asked a total of 5400 women to respond concerning LUTS (Overactive Bladder Symptom Score, OABSS; International Consultation on Incontinence Questionnaire‐Short Form, ICIQ‐SF) and their feelings of depression (Quick Inventory of Depressive Symptomatology‐Japanese version, QIDS‐J). Of these individuals, 4151 (76.9%) answered the questionnaire completely. We also collected data on respondents' age, household income, type of residency, occupation, marital status, and number of children.

The OABSS, originally developed in Japan, is a 4‐item questionnaire that expresses OAB symptoms on a single scale.^12^ The OABSS question items address the following individual symptoms: daytime frequency, nocturia, urgency, and urgency incontinence. Gotoh et al. reported that the OABSS was useful for assessing the effects of treatment on OAB symptoms and was responsive to treatment‐related changes.^13^ The OABSS consists of the following four questions (Q) regarding specific symptoms: daytime frequency (Q1), night‐time frequency (Q2), urgency (Q3), and urgency incontinence (Q4). The OABSS was defined as the sum of the total OABSS, with OAB defined as the presence of both a total score of ≥3 and an OABSS Q3 score of ≥2. The OABSS defined urgency urinary incontinence (UUI) as an OABSS Q4 score of ≥1. Daytime frequency was defined as an OABSS Q1 score of ≥1. Nocturia was defined as an OABSS Q2 score of ≥2. The ICIQ‐SF was developed to screen for incontinence; obtain a brief yet comprehensive summary of the level, impact, and perceived causes of symptoms of incontinence; and facilitate patient–clinician discussions.^14^ The ICIQ‐SF score was calculated as the sum of the Q1, Q2, and Q3 scores. UUI (ICIQ‐SF definition) was defined by a positive response to “leaks occur before you can get to the toilet.” SUI was defined by a positive response to at least one of the following questions: “leaks occur when you cough or sneeze” and “leaks occur when you are physically active/exercising.” Mixed urinary incontinence was defined as both UUI (ICIQ‐SF definition) and SUI. Post‐micturition dribble was defined as a positive response to “leaks occur when you have finished urinating and are dressed.”

The QIDS‐J was translated and validated from the QIDS‐Self Report (QIDS‐SR), which consists of 16 questions and plays a role in the diagnostic criteria for major depressive disorder. These questions were divided into nine categories. Four concerned sleeping disorders, four appetite and body weight, two psychological disorders, and the remaining nine other topics. The final score was calculated as the sum of the highest points for sleeping disorders, appetite and body weight, and psychological disorders and the sum of six questions.^15^

### 2.1 Statistical analyses

The participants' characteristics and scores were analyzed by the *t* test and chi‐square test. A one‐factor analysis of variance was used to compare the QIDS‐J score and urinary symptoms using the Graph Pad Prism software program (Graph Pad Software; La Jolla, California). *p* values of <.05 were considered to indicate statistical significance.

## 3 RESULTS

Responses concerning urinary symptoms in 4151 Japanese women were obtained. Individual characteristics are shown in Table 1, including marital status, presence of children, household income, occupation, and age. A total of 2666 of the 4151 (64.2%) respondents were married, and 2189 (52.7%) had at least one child. The number (%) with a certain household income was 1756 (42.3%) making under JPY4 000 000/year, 1613 (38.9%) making JPY4 000 000–8 000 000/year, 555 (23.4%) making JPY8 000 000–12 000 000/year, and 154 (3.7%) making over JPY12 000 000/year. The distribution of each questionnaire (OABSS<ICIQ‐SF, and QIDS‐J) score is shown in Figure S1.

**TABLE 1:** Patients' background characteristics.

Variables	Median (mean +/− SD), *n* (%)
Number of patients	4151
Age (years)	48 (48.3 +/− 13.8)
Marriage (yes)	2666 (64.2%)
Children (yes)	2189 (52.7%)
Household income	
Less than JPY4 000 000	1756 (42.3%)
JPY4 000 000–8 000 000	1613 (38.9%)
JPY8 000 000–12 000 000	555 (13.4%)
More than JPY12 000 000	154 (3.7%)

**FIGURE 1:**
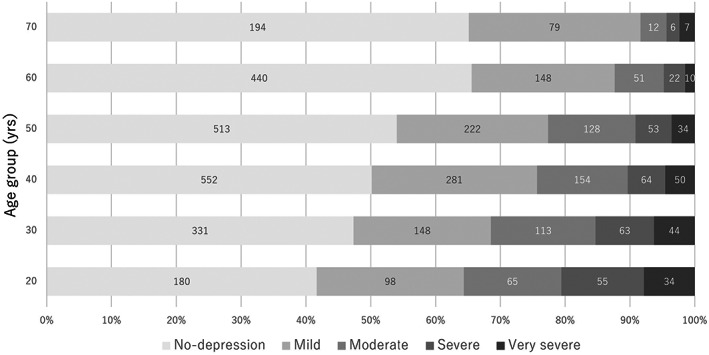
Quick Inventory of Depressive Symptomatology‐Japanese version (QIDS‐J) group distribution in each generation.

The median (mean ± SD) age was 48 (48.3 ± 13.8) years. The overall prevalence of OAB was 14.2% and that of UUI 20.3%. Figure 1 shows the depression status determined using QIDS‐J for each generation. In this cohort, the young generation (20‐ to 29‐year‐olds) showed the highest ratio of severe and very severe depression (12.7% and 7.9%, respectively) among respondents.

**FIGURE 2:**
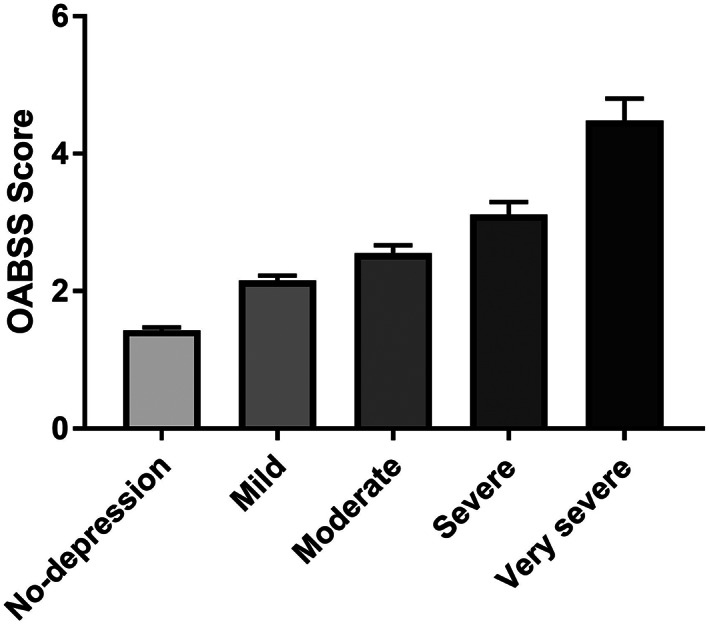
OABSS in each Quick Inventory of Depressive Symptomatology‐Japanese version (QIDS‐J) score group. OABSS, Overactive Bladder Symptom Score.

**FIGURE 3:**
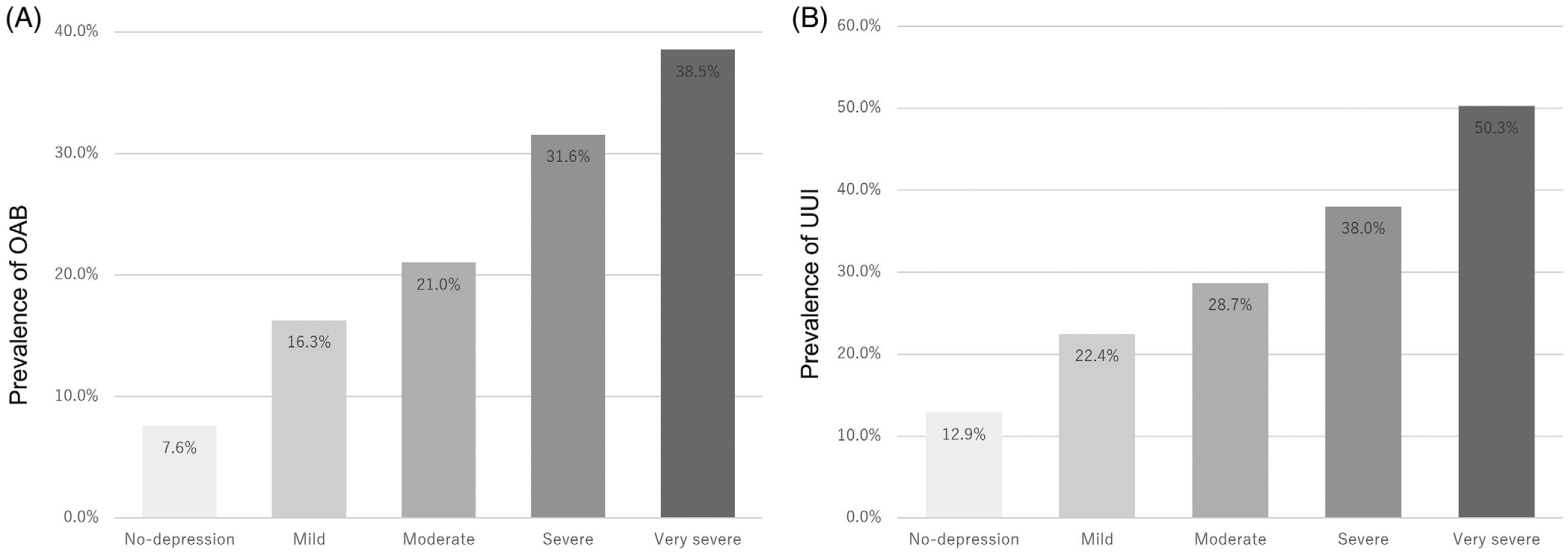
Prevalence of (A) OAB and (B) UUI in each Quick Inventory of Depressive Symptomatology‐Japanese version (QIDS‐J) score group. OAB, overactive bladder; UUI, urgency urinary incontinence.

**FIGURE 4:**
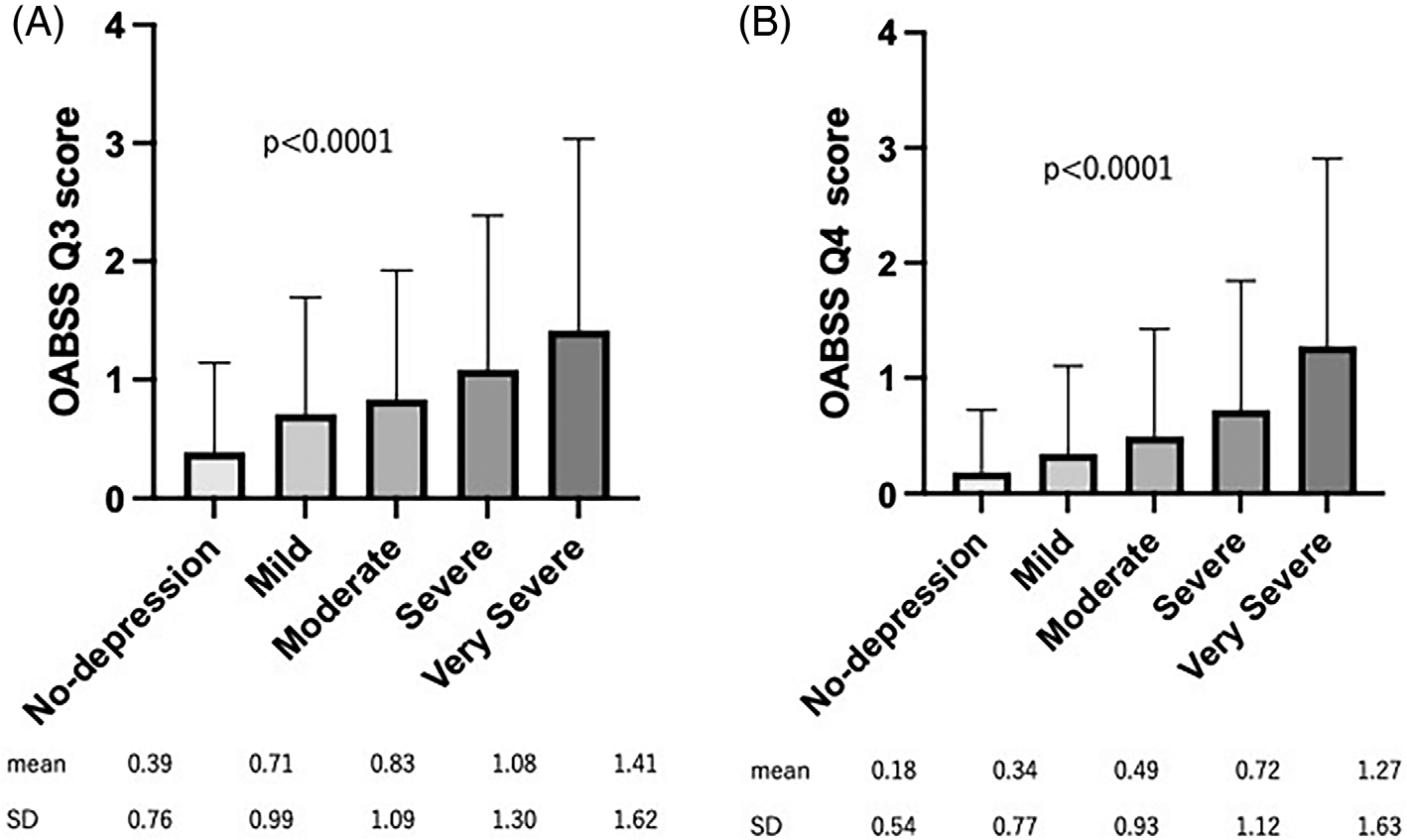
OABSS Q3 (urgency) and Q4 (urgency incontinence) in each Quick Inventory of Depressive Symptomatology‐Japanese version (QIDS‐J) score group. OABSS, Overactive Bladder Symptom Score.

In terms of the correlation between the depression status and OAB symptoms, the OABSS was positively correlated with the QIDS‐J score (*p* < .001) (Figure 2). The mean ± SD OABSS was 1.43 ± 1.76 in the normal group, 2.16 ± 2.22 in the mild group, 2.55 ± 2.58 in the moderate group, 3.11 ± 30.5 in the severe group, and 4.49 ± 4.44 in the very severe group. The prevalence of OAB and UUI in each QIDS‐J score group increased along with the severe QIDS‐J score (Figure 3A,B). The respective prevalence of OAB and UUI was 7.6% and 12.9% in the normal group, 16.3% and 22.4% in the mild group, 21.0% and 28.7% in the moderate group, 31.6% and 38.0% in the severe group, and 38.5% and 50.3% in the very severe group. In terms of urgency and urgency incontinence, urgency (OAB Q3 score) and urgency incontinence (OAB Q4 score) were positively correlated with depression status (Figure 4).

In all age groups, the prevalence of OAB and UUI was positively correlated with the depression status (Table 2). In the young subjects (20–39 years old), the respective relative risk (RR) of OAB and UUI was 5.77 and 5.38 in the QIDS‐J severe group and 7.42 and 7.44 in the QIDS‐J very severe group. In contrast, for those 60–80 years old, the respective RR of OAB and UUI was 3.02 and 2.47 in the QIDS‐J severe group and 3.73 and 3.06 in the QIDS‐J very severe group. The influence of depression on LUTS was lower in the elderly group (60–80 years old) than in the other groups.

**TABLE 2:** Correlation between QIDS‐J group and prevalence of OAB and UUI in each generation.

Age group	No. of individuals	OAB positive (cases, %)		RR	UUI positive (cases, %)		RR
20–39	1131	179	15.8%		201	17.8%	
No depression	511	30	5.9%	Reference	37	7.2%	Reference
Mild	246	37	15.0%	2.56	34	13.8%	1.91
Moderate	178	38	21.3%	3.64	42	23.6%	3.26
Severe	118	40	33.9%	5.77	46	39.0%	5.38
Very severe	78	34	43.6%	7.42	42	53.8%	7.44
40–59	2051	281	13.7%		416	20.3%	
No depression	1065	78	7.3%	Reference	138	13.0%	Reference
Mild	503	78	15.5%	2.12	112	22.3%	1.72
Moderate	282	61	21.6%	2.95	85	30.1%	2.33
Severe	117	35	29.9%	4.08	42	35.9%	2.77
Very severe	84	29	34.5%	4.71	39	46.4%	3.58
60–80	969	129	13.3%		227	23.4%	
No depression	634	60	9.5%	Reference	110	17.4%	Reference
Mild	227	44	19.4%	2.05	73	32.2%	1.85
Moderate	63	11	17.5%	1.84	23	36.5%	2.10
Severe	28	8	28.6%	3.02	12	42.9%	2.47
Very severe	17	6	35.3%	3.73	9	52.9%	3.05
Total	4151	589	14.2%		844	20.3%	

Abbreviations: OAB; overactive bladder; QIDS‐J, Quick Inventory of Depressive Symptomatology‐Japanese version; RR, relative risk; UUI, urgency urinary incontinence.

## 4 DISCUSSION

This study is the first to examine the correlation between depression status and LUTS in women using a large cohort of approximately 4100 Japanese women. Some studies have suggested that depression worsens urinary symptoms, particularly nocturia.^10^, ^11^

The QIDS‐J score was relatively high in young subjects and gradually decreased with age (Figure 1). Weinberger et al. reported the prevalence of depression in the United States from 2005 to 2015. In their study, the total number of patients gradually decreased over time. The 12‐ to 17‐year‐olds showed the highest prevalence of depression, and this prevalence decreased with age, with those ≥50 years old showing the lowest prevalence of depression.^12^ Around 6% of humans have major depressive disorder (MDD), and individuals with MDD are three times more likely to have depression than those without MDD.^13^, ^14^ More than 40% experience their initial depressive episode before the age of 20 and progress to depression around the age of 25.^15^ Depression is twice as prevalent in women as in men. The detailed mechanism underlying the higher prevalence of depression in women than in men is unknown, but the involvement of hormones or neurogenesis has been suspected.^16^

The mean OABSS increased as the QIDS‐J score increased. Previous reports have revealed that LUTS reduces patients' daily quality of life.^2^, ^17^, ^18^, ^19^ Skalski et al. reported that a moderate or worse depressive score on the QIDS‐SR was correlated with a higher International Prostate Symptom Score (IPSS), and LUTS was correlated with depressive symptoms.^17^ Lee et al. investigated a total of 8284 Asian individuals to determine the correlation between LUTS and quality of life using the OABSS, IPSS, International Continence Society, and Hospital Anxiety and Depression Scale scores, and the severe depression group showed a higher ratio of having LUTS than other groups.^20^ Coyne et al. suspected that LUTS were correlated with a reduced health‐related quality of life and thus related to depression and anxiety.^21^

Our results suggest that depression caused endothelial sclerosis and a worsened bladder function, especially in the young group. Recent studies have shown that depression worsens endothelial sclerosis.^22^ Phosphodiesterase type 5 (PDE‐5) inhibitors inhibit PDE‐5, which is distributed in the vascular endothelium and smooth muscles of the bladder, and also increase the concentration of cyclic guanosine monophosphate produced in response to the level of nitric oxide in tissue.^23^ Based on these mechanisms, the blood flow and oxygen supply increase in tissue, resulting in an improvement in the blood flow of the lower urinary tract.^23^ The present and previous findings suggest that endothelial sclerosis has a greater impact on LUTS in young LUTS patients than in elderly patients.

Several limitations associated with the present study warrant mention. First, this study examined the depression status based on the QIDS‐J score, not as diagnosed by a psychiatrist. Given that we were investigating a large cohort, we had to use a self‐administered questionnaire to evaluate the depression score. However, both the QIDS‐J and OABSS are language‐validated symptom scores widely used in daily clinical practice to evaluate depression status and urinary symptoms. Second, this study did not evaluate other risk factors. Previous reports have shown that malignancy, gynecological surgical history, diabetes mellitus, hypertension, and smoking history are potential risk factors for worsened urinary symptoms, as are obesity, diet, dyslipidemia, hormonal imbalance, and alcohol, which were ignored in this study.^24^, ^25^, ^26^, ^27^ The percentage of subjects with these risk factors was shown to be positively correlated with aging, with younger generations who had fewer of these risk factors showing a higher RR for OAB and UUI with depression than other generations. Further studies are needed to clarify the influence of other risk factors. Third, this study determined OAB and UUI solely by a web‐based questionnaire, with no other evaluations, such as a urinary analysis, conducted. Future studies should be conducted on patients who were diagnosed with depression, collecting more clinical information from the subjects.

In conclusion, this study revealed that worsening LUTS was correlated with depression. The RR of OAB and UUI influenced by depression was higher in younger groups than in older groups.

## CONFLICT OF INTEREST

We declare no conflicts of interests.

## ETHICS STATEMENT

This study has been approved by the institutional review board of Yokohama City University Medical Center (approval no. B201000052).

## INFORMED CONSENT

Following an institutional review board‐approved protocol, informed consent was obtained from the participants before answering the questionnaires.

## Supporting information


**Figure S1.** The number of patients with each questionnaire's score: (A) OABSS, (B) ICIQ‐SF, and (C) QIDS‐J.

## Data Availability

Research data are not shared.
