# Supplementary figures and images for: Lower urinary tract symptoms are elevated with depression in Japanese women
Source: Low Urin Tract Symptoms. 2023 Mar 30;15(4):116–21. doi: 10.1111/luts.12478 (PMC11500686; doi:10.1111/luts.12478)

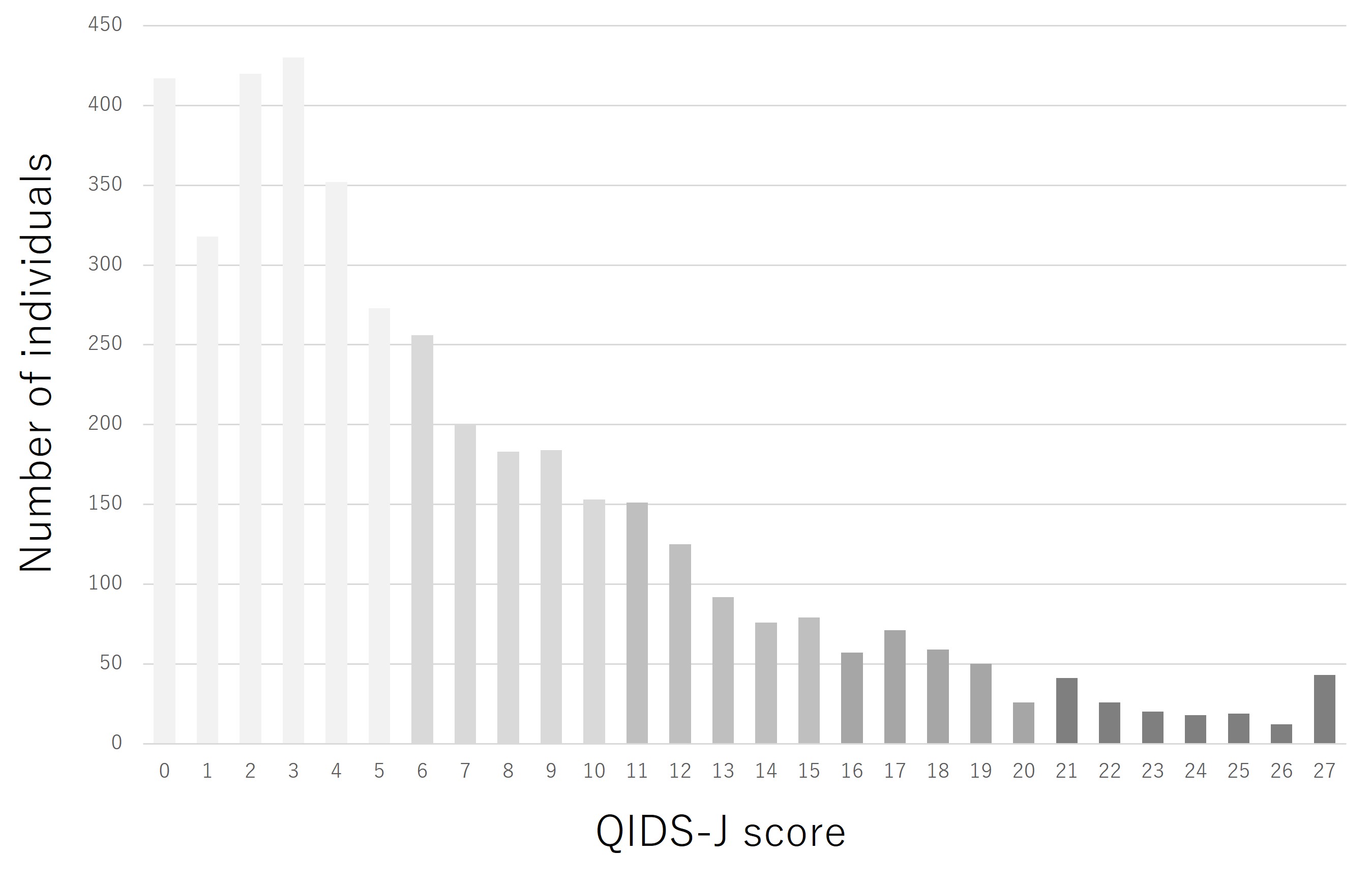

Supplement: Supplementary file 1 — Figure S1. The number of patients with each questionnaire's score: (A) OABSS, (B) ICIQ‐SF, and (C) QIDS‐J. [file LUTS-15-116-s001.jpg]
